# Microbial nitrification in throughfall of a Japanese cedar associated with archaea from the tree canopy

**DOI:** 10.1186/s40064-016-3286-y

**Published:** 2016-09-17

**Authors:** Keiji Watanabe, Ayato Kohzu, Wataru Suda, Shigeki Yamamura, Takejiro Takamatsu, Akio Takenaka, Masami Kanao Koshikawa, Seiji Hayashi, Mirai Watanabe

**Affiliations:** 1 National Institute for Environmental Studies, 16-2 Onogawa, Tsukuba, Ibaraki 305-8506 Japan; 2 Center for Environmental Science in Saitama, Kazo, Saitama 347-0115 Japan; 3 Graduate School of Frontier Science, The University of Tokyo, Kashiwa, Chiba 277-8562 Japan

**Keywords:** Ammonia-oxidizing archaea, *amoA*, Throughfall, Nitrification, Phyllosphere

## Abstract

To investigate the nitrification potential of phyllospheric microbes, we incubated throughfall samples collected under the canopies of Japanese cedar (*Cryptomeria japonica*) and analyzed the transformation of inorganic nitrogen in the samples. Nitrate concentration increased in the unfiltered throughfall after 4 weeks of incubation, but remained nearly constant in the filtered samples (pore size: 0.2 and 0.4 µm). In the unfiltered samples, δ^18^O and δ^15^N values of nitrate decreased during incubation. In addition, archaeal ammonia monooxygenase subunit A (*amoA*) genes, which participate in the oxidation of ammonia, were found in the throughfall samples, although betaproteobacterial *amoA* genes were not detected. The *amoA* genes recovered from the leaf surface of *C. japonica* were also from archaea. Conversely, nitrate production, decreased isotope ratios of nitrate, and the presence of *amoA* genes was not observed in rainfall samples collected from an open area. Thus, the microbial nitrification that occurred in the incubated throughfall is likely due to ammonia-oxidizing archaea that were washed off the tree canopy by precipitation.

## Background

The phyllosphere is a hostile environment for microbes because the leaf surface is deficient in nutrients and exposed to dry conditions and strong UV radiation (Yang et al. 2001). Nevertheless, bacteria exist in the phyllosphere at an estimated average density of 10^6^–10^7^ cells cm^−2^ (Lindow and Brandl 2003). The total leaf surface area of terrestrial plants is estimated to be 6.4 × 10^8^ km^2^, which is comparable to Earth’s terrestrial surface area (Morris and Kinkel 2002). Thus, if phyllospheric microbes metabolize nutritional elements (e.g., N and S), they may play an important role in the biogeochemical cycles of these elements. Phyllospheric microbes have been well studied and include plant pathogens and their antagonists, ice-nucleation bacteria, decomposers, phytohormone producers, and nitrogen fixers (Fett et al. 1987; Hirano and Upper 2000; Enya et al. 2007; Fürnkranz et al. 2008; Suda et al. 2009; Vorholt 2012). However, there is limited information available regarding the contribution of phyllospheric microbes to elemental dynamics (e.g., Woods et al. 2012).

Nitrogen is an abundant element that is essential to living organisms. Microbes drive biogeochemical cycling processes for nitrogen, including N-fixation, N-mineralization, N-immobilization, denitrification, and nitrification (Francis et al. 2007; Gruber and Galloway 2008; Hayatsu et al. 2008; Ollivier et al. 2011). Nitrification is a two-step process. In the first step, ammonia is oxidized to nitrite by ammonia-oxidizing archaea (AOA) and/or ammonia-oxidizing bacteria (AOB). In the second step, nitrite is oxidized to nitrate by nitrite-oxidizing bacteria (NOB). Recent studies have revealed that AOA and AOB are widely distributed in soil, marine, and freshwater environments, and that they may contribute to the global nitrogen cycle (Leininger et al. 2006; Agogué et al. 2008; Prosser and Nicol 2008; Herrmann et al. 2009; Martens-Habbena et al. 2009; Church et al. 2010). Furthermore, separation of the ecological niches of AOA and AOB has been observed in soil and marine environments (Erguder et al. 2009; Di et al. 2009; Nunoura et al. 2015). The leaf surface of trees constantly receives ammonium ions and ammonia via atmospheric deposition; accordingly, the phyllosphere might be a suitable habitat for some species of ammonia-oxidizing microbes (Shi et al. 2014; Guerrieri et al. 2015). Two previous studies employing the most probable number (MPN) and fluorescence in situ hybridization (FISH) techniques have suggested the presence of ammonia-oxidizing microbes in the phyllosphere (Papen et al. 2002; Teuber et al. 2007). However, evidence of the potential for microbial ammonia oxidation in the tree phyllosphere has not been thoroughly verified.

It is very likely that throughfall (rainwater that drips from tree canopies) contains phyllospheric microbes washed off from the canopies, as well as airborne substances and secretions from plants and microbes (Müller et al. 2004; Gaige et al. 2007). Therefore, if phyllospheric microbes possess nitrifying ability, transformation of nitrogen (from NH_4_^+^ to NO_3_^−^) will occur in the throughfall when it is incubated under appropriate conditions. Recently, analysis of isotopic (δ^18^O, ∆^17^O, and δ^15^N) changes in nitrate throughout the rainfall, throughfall, and stemflow samples provided evidence of microbial nitrification in the tree canopy (Shi et al. 2014; Guerrieri et al. 2015).

In this study, throughfall samples collected under the canopies of Japanese cedar (*Cryptomeria japonica*), which is an evergreen conifer planted widely across Japan, were incubated to examine nitrate production (nitrification) in the samples. Because mixing of nitrate produced by microbial activity (nitrification) can decrease the δ^18^O value of nitrate in throughfall samples (Kendall 1998; Xue et al. 2009; Snider et al. 2010; Shi et al. 2014; Guerrieri et al. 2015), we also measured the δ^18^O changes in nitrate during incubation. In addition, the presence of the ammonia monooxygenase subunit A (*amoA*) gene, which is a functional gene that participates in the oxidation of ammonia by ammonia-oxidizing microbes, was determined to characterize the relative contributions of AOA and AOB to nitrification in the throughfall. The results were then compared with those obtained from sterile (filtered) throughfall samples and rainfall samples to show that nitrification was mainly due to phyllospheric microbes. Furthermore, the leaf surfaces of *C. japonica* were analyzed for the presence of *amoA* genes to discuss the possibility of the existence of ammonia-oxidizing microbes in the phyllosphere.

## Methods

### Sampling site

Sampling was conducted at the National Institute for Environmental Studies (NIES; 36°03′N, 140°07′E) in Tsukuba, Ibaraki, Japan. Tsukuba is a suburban city with a population of 215,000 within 284 km^2^ that is located about 50 km northeast of metropolitan Tokyo and 40 km west of the Pacific Ocean. The mean annual temperature, relative humidity, wind velocity, and annual precipitation at NIES between 2006 and 2009 were 14.8 °C, 71 %, 1.6 m s^−1^, and 1178 mm, respectively (data from the Air Quality Research Station of NIES). Throughfall and leaf samples were collected from two (Cj1 and Cj2) and three (Cj1, Cj2, and Cj3) isolated *C. japonica* trees, respectively. The diameter of the trunk at breast height of Cj1, Cj2, and Cj3 was 42, 42, and 47 cm, respectively. These trees had abundant foliage and were approximately 200–500 m apart. Rainfall unaffected by the tree canopies was also collected from an open grassplot approximately 100 m from the nearest throughfall collection sites.

### Throughfall and rainfall sampling

**Fig. 1:**
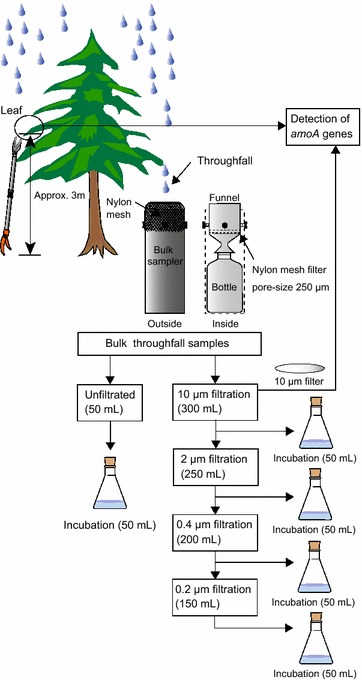
Experimental flow of throughfall collection, filtration treatment prior to incubation, and sample collection for the detection of *amoA* genes from filters and leaves

Throughfall was collected under the canopies of Cj1 and Cj2 from 22 February to 22 March 2006. Sampling was conducted using a bulk sampler as previously described (Hou et al. 2005). Throughfall collected by a polyethylene funnel (10 cm in diameter) was stored in a 2-L polyethylene bottle (Fig. 1). A nylon mesh filter was placed at the bottom of the funnel to minimize contamination by insects and plant debris. The funnel and a bottle were put in an opaque PVC tube to keep the sample in the dark and prevent growth of algae. The apparatus was washed successively with alkaline detergent solution and acidic solution, then rinsed thoroughly with deionized water before use. The funnel was placed 1 m above the ground, 50–100 cm from tree trunks, under the canopies with the highest leaf density. Rainfall sampling was conducted concurrently with throughfall collection using the same method.

### Filtration and incubation of throughfall and rainfall samples

Immediately after the collection, throughfall and rainfall samples were sequentially filtered to remove suspended solids (SS) and microbes using Nuclepore membrane filters (Whatman, Middlesex, UK) with pore sizes of 10 (to remove large SS and aggregated microbes), 2 (to remove medium SS and large microbes), 0.4 (to remove small SS and most microbes), and 0.2 µm (to remove very small SS and large viruses) (Fig. 1). All unfiltered and filtered samples (50 mL in each fraction) were dispensed into sterile 50-mL Erlenmeyer flasks with foam silicon stoppers and incubated at 30 °C in the dark. Following incubation periods of 1, 2, 3, and 4 weeks, nitrate, nitrite, and ammonium ions in each sample were analyzed using ion chromatography (DX-100) with an IonPack AS12A/AG12A and IonPack CS3/CG3 column (Dionex, Sunnyvale, CA, USA). Analysis of ammonium ions was only conducted for samples before incubation and after incubation for 1 and 4 weeks. The filtrates and filters were stored at −20 °C until further analysis.

### Analysis of stable isotopic ratios (δ^18^O and δ^15^N) of nitrate in throughfall and rainfall samples

The oxygen and nitrogen isotope ratios (δ^18^O and δ^15^N) of nitrate were measured by the denitrifier method developed by Sigman et al. (2001) and Casciotti et al. (2002) after removal of nitrite by the addition of ascorbic acid (Granger et al. 2006). This method enables the measurement of δ^18^O and δ^15^N in small amounts of nitrate (i.e., 30 nmol). In this method, N_2_O gas converted from nitrate catalytically by cultured denitrifying bacteria (*Pseudomonas chlororaphis* subsp. *aureofaciens* ATCC 13985) was introduced into an isotope ratio mass spectrometer equipped with a custom-made auto-injection preprocessing system for N_2_O gas (Kohzu et al. 2011). Stable isotope ratios are expressed in δ notation as the difference in parts per thousand (‰) from the standard (i.e., δ^18^O = 0 ‰ in Vienna Standard Mean Ocean Water; δ^15^N = 0 ‰ in atmospheric N_2_):

$$\begin{aligned} &\updelta^{18} {\text{O}} = ({\text{R}}\,{\text{sample/R}}\,{\text{standard}} - 1) \times 1000,\quad {\text{where}}\,{\text{R}}\,{\text{is}}\,^{18} {\text{O}}/^{16} {\text{O}}. \\ &\updelta^{15} {\text{N}} = ({\text{R}}\,{\text{sample/R}}\,{\text{standard}} - 1) \times 1000,\quad {\text{where}}\,{\text{R}}\,{\text{is}}\,^{15} {\text{N}}/^{14} {\text{N}}. \\ \end{aligned}$$

Four internal standards (IAEA-N3, USGS32, USGS34, and USGS35) were used to correct the measured values. The analytical precision was ±0.59 and ±0.19 ‰ for δ^18^O and δ^15^N, respectively. The isotope ratios (δ^18^O and δ^15^N) of nitrate in rainfall samples were only measured for the samples before incubation and for unfiltered samples after 4 weeks of incubation.

### Extraction of microbial DNA in throughfall and rainfall samples

The membrane filters used for filtration (described above) were subjected to the extraction of microbial genomic DNA (Fig. 1). The extraction was conducted directly for every filter sample prepared using a FastDNA SPIN kit for Soil (Q-Biogene, Carlsbad, CA, USA) according to the manufacturer’s instructions. It should be noted that a clone library was constructed for archaeal *amoA* genes obtained from the 10-µm-pore filter of throughfall for Cj1. Details regarding the genetic analyses are described below.

### Leaf sampling and extraction of microbial DNA from leaves

Small branches of *C. japonica* (Cj1, Cj2, and Cj3) were collected from an accessible height of approximately 3 m above the ground on 19 March 2010 using long reach pruning shears while preventing branches from falling on the ground (Fig. 1). The collected branches were placed in clean polyethylene bags without touching with bare hands and brought to the laboratory, after which leaves about 5 cm in length with small stems were separated from the branches without distinction of leaf age and used for extraction of microbial genomic DNA. Leaf samples (10 g) were placed into 50-mL sterile polypropylene centrifuge tubes (Greiner Bio-one, Frickenhausen, Germany), after which 30–40 mL of sterile potassium phosphate buffer (pH 7.0) was added. Microbial cells from the leaves were collected by ultrasonic disintegration (45 kHz, 10 min) and centrifugation (5800×*g*, 10 min, 5 °C), after which the DNA for genetic analyses was extracted from the precipitates using a FastDNA SPIN kit for Soil (Q-Biogene) according to the manufacturer’s instructions. All sampling and DNA extraction processes were conducted while wearing a clean pair of nitrile rubber gloves.

### Detection of archaeal and betaproteobacterial *amoA* genes

Microbial DNA obtained from throughfall and rainfall samples and from leaves of *C. japonica* was subjected to genetic analysis as follows. Partial archaeal and betaproteobacterial *amoA* genes were amplified by polymerase chain reaction (PCR) using either archaeal or betaproteobacterial *amoA* gene-specific primers. Specifically, amoA19F (Leininger et al. 2006) and amo643R (Treusch et al. 2005) were used for the archaeal *amoA* gene, while amoA-1F and amoA-2R (Rotthauwe et al. 1997) were used for the betaproteobacterial *amoA* gene (fungal genes were not analyzed). The reaction was performed in a 50-µL mixture containing 0.1–2 ng of template DNA, 0.2 µM of each primer, 5 µL of 10× Ex Taq buffer, 4 µL of dNTP mixture, 1 µL of bovine serum albumin (20 mg mL^−1^; Takara Bio Inc., Shiga, Japan), and 0.25 µL of TaKaRa Ex Taq HS (Takara Bio Inc., Shiga, Japan). The template DNA concentration was measured using a Qubit 2.0 fluorometer and a Quant-iT dsDNA HS assay kit (Thermo Fisher Scientific, Waltham, MA, USA) in accordance with the manufacturer’s instructions. The thermal cycling conditions were as follows for the archaeal *amoA* gene: 95 °C for 10 min, followed by 36–42 cycles of 95 °C for 1 min, 55 °C for 1 min and 72 °C for 40 s and then final extension at 72 °C for 10 min. For the betaproteobacterial *amoA* gene, the cycling profile was 95 °C for 10 min, followed by 45 cycles consisting of 95 °C for 1 min, 55 °C for 1 min and 72 °C for 40 s, and then final extension at 72 °C for 10 min. The production of both amplicons was confirmed by 2 % agarose gel electrophoresis.

### Quantification of archaeal *amoA* genes by quantitative real-time PCR (qPCR)

Partial archaeal *amoA* genes detected in the throughfall and leaf samples were quantified by qPCR (LightCycler Nano; Roche, Basel, Schweitz) using archaeal *amoA* gene-specific primers (amoA19F and amo643R). The reaction was conducted in a 20-µL reaction mixture containing 3.8 µL of distilled H_2_O, 5 µL containing 0.1–2 ng of template DNA, 0.4 µL of 10 µM each primer, 0.4 µL of bovine serum albumin (20 mg mL^−1^; Takara Bio Inc.), and 10 µL of 2× SYBR Premix Ex Taq (Tli RNaseH Plus) (Takara Bio Inc.). The template DNA concentration was measured using a Qubit 2.0 fluorometer and a Quant-iT dsDNA HS assay kit (Thermo Fisher Scientific) in accordance with the manufacturer’s instructions. The thermal cycling conditions were as follows for the archaeal *amoA* gene: 95 °C for 30 s; 45 cycles consisting of 95 °C for 30 s, 55 °C for 1 min and 72 °C for 40 s; final extension at 72 °C for 10 min with a melting curve analysis.

The archaeal *amoA* gene was amplified from an environmental clone, N3-16 (AB622272), using the amoA19F and amo643R primers. The PCR products were purified using a QIAquick PCR purification kit (Qiagen). This amplicon was used for construction of a standard curve of qPCR. qPCR analysis of each sample was conducted in triplicate, and the efficiency of the qPCR reaction was 86.6–87.5 %. The quantitative lower limit for the archaeal *amoA* gene was 44 copies per reaction.

### Cloning and sequence analysis of archaeal *amoA* Genes

PCR-amplified fragments of archaeal *amoA* gene were cloned using the pGEM-T Easy Vector System with DH5α *Escherichia coli* (Promega, Madison, WI, USA) in accordance with the manufacturer’s instructions. The cloned DNA was amplified by PCR using T7 and SP6 promoter primers (Promega). The PCR products were purified using a MinElute 96 UF PCR Purification Kit (Qiagen, Hilden, Germany) and used as templates for sequencing. Sequencing was performed with the amoA19F and amo643R primer set using a BigDye Terminator v3.1 Cycle Sequencing Kit (Applied Biosystems, Carlsbad, CA, USA) and an ABI PRISM 3100 genetic analyzer (Applied Biosystems).

The obtained sequences were compared with known references using BLAST searches of the DNA Data Bank of Japan (DDBJ; http://www.ddbj.nig.ac.jp). Sequence data were aligned using the ClustalW package (Thompson et al. 1994), and phylogenetic trees were constructed using the neighbor-joining method (Saitou and Nei 1987) with the Poisson model in the MEGA 6.0 software package (Tamura et al. 2013). Bootstrap re-sampling analysis (1000 replicates) was performed to estimate the confidence of tree topologies (Felsenstein 1985). The nucleotide sequences of partial archaeal *amoA* genes were deposited into the DDBJ database under the following accession numbers: AB622267–AB622275, AB649997–AB650012, and AB873108–AB873179.

## Results

### Nitrate production in throughfall and rainfall samples

**Fig. 2:**
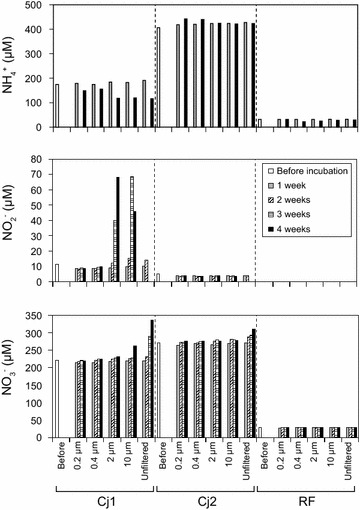
Changes in ammonium, nitrite, and nitrate concentrations in the incubated throughfall samples of Cj1 and Cj2 and rainfall samples (RF), which were passed through several filters with different pore sizes before incubation. Ammonium concentrations in samples incubated for 2 and 3 weeks were not determined

Overall, 70 and 48 mm of throughfall were collected from under the canopies of Cj1 and Cj2, respectively, from 22 February to 22 March 2006, while 75 mm of rainfall were collected. The initial concentrations of mineral nitrogen in the throughfall from Cj1 (404 µM) and Cj2 (677 µM) were higher than in the rainfall (59 µM). The initial ammonium concentration was about twofold lower in the throughfall from Cj1 (174 µM) than Cj2 (404 µM) (Fig. 2). Although the initial nitrite concentration was higher in Cj1 throughfall (11 µM) than Cj2 throughfall (5 µM), the nitrate concentrations were nearly equivalent in Cj1 (219 µM) and Cj2 (267 µM).

For throughfall from Cj1 (Fig. 2), nitrate concentrations in the unfiltered sample increased during 4 weeks of incubation, whereas there was almost no nitrate production in samples passed through the 0.2-, 0.4-, and 2-µm-pore filters. Ammonium decreased in all samples during 4 weeks of incubation, although the decreases were larger in the unfiltered samples and those that passed through 2- and 10-µm-pore filters. Conversely, nitrite was not detected in the unfiltered samples after 3 and 4 weeks of incubation, but an increase in nitrite concentration was observed in samples passed through the 2- and 10-µm-pore filters after 3 and 4 weeks of incubation. For throughfall from Cj2, almost no transformation of inorganic nitrogen was observed, but nitrate increased slightly and nitrite disappeared during incubation of the unfiltered sample. In contrast, nitrate and nitrite were not produced in any rainfall samples, regardless of the presence or absence of filtration, although the initial ammonium concentration was quite low.

### Changes in δ^18^O and δ^15^N of nitrate in throughfall and rainfall samples during incubation

**Fig. 3:**
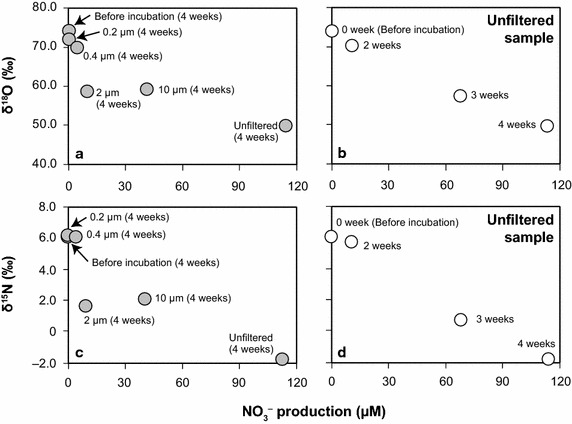
Nitrate production and isotopic ratios (δ^18^O and δ^15^N) of nitrate in throughfall samples from Cj1 passed through 0.2-, 0.4-, 2-, and 10-µm-pore filters after 4 weeks of incubation (**a** δ^18^O, **c** δ^15^N), and those in the unfiltered samples after different incubation periods (**b** δ^18^O, **d** δ^15^N). Unfiltered samples incubated for 1 week were not analyzed. The data points of “before incubation (**a**, **c**)” and “0 weeks (**b**, **d**)”, and those of “unfiltered (**a**, **c**)” and “4 weeks (**b**, **d**)” are same meaning

For the throughfall sample from Cj1, decreases in δ^18^O and δ^15^N values of nitrate were observed when it was incubated for 4 weeks without filtration or after filtration through 2- and 10-µm-pore filters (Fig. 3a, c), although such isotopic changes were small or not observed in samples passed through 0.2- and 0.4-µm-pore filters. In the unfiltered samples from Cj1, a gradual decrease of δ^18^O and δ^15^N values of nitrate that coincided with nitrate production occurred with increasing incubation time (Fig. 3b, d). For throughfall samples from Cj2, even in the unfiltered samples, the decreases in δ^18^O (from +75.2 to +71.3 ‰) and δ^15^N (from +6.1 to +4.0 ‰) values were relatively small during the 4 weeks of incubation because nitrate production was limited (Fig. 2). In addition, the δ^18^O and δ^15^N values of nitrate in unfiltered rainfall samples did not change before and after incubation, being approximately +62 and +0.5 ‰, respectively.

### Archaeal and betaproteobacterial *amoA* genes in throughfall and on tree leaves

**Fig. 4:**
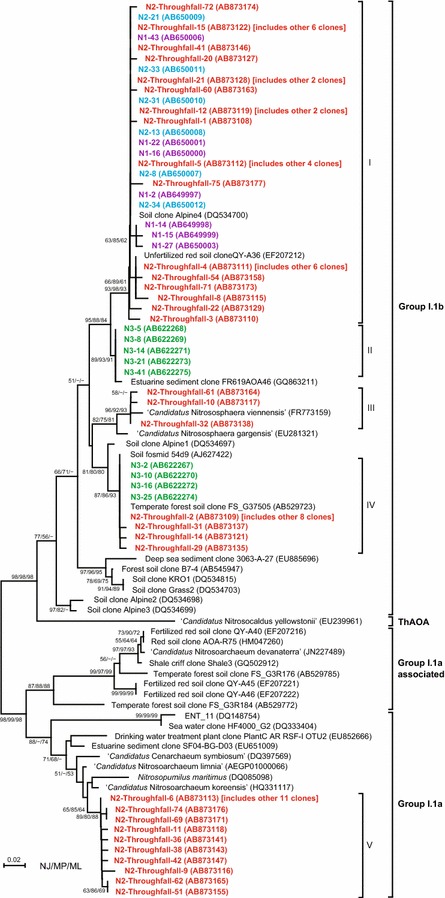
Phylogenetic tree generated using the neighbor-joining method to analyze 178 deduced amino acid sequences of archaeal *amoA*-like genes from microbes trapped in the 10-µm-pore filter for throughfall of Cj1 (*red* clone name N2-throughfall, AB873108–AB873179) and phyllospheric microbes on the leaves of *C. japonica* [Cj1 (*blue* clone name N2, AB650007–AB650012), Cj2 (*green* clone name N3, AB622267–AB622275), and Cj3 (*purple* clone name N1, AB649997–AB650012)]. The *bar* represents a 2 % evolutionary distance, and bootstrap values above 50 % are shown. *Node percentages* represent the bootstrap values based on neighbor-joining (NJ), maximum parsimony (MP), and maximum likelihood (ML)

The filters used for throughfall samples were analyzed for *amoA* genes by PCR because they should have contained the microbes washed off from the tree canopies. Archaeal *amoA*-like gene amplicons were detected from 0.4- to 10-µm-pore filters used for throughfall from Cj1 and Cj2, whereas betaproteobacterial *amoA* gene amplicons were not detected from those filters. Conversely, the filters used for rainfall contained neither archaeal nor betaproteobacterial *amoA* genes. qPCR analysis revealed that the abundance of archaeal *amoA* genes on the 10-µm-pore filter, through which 300 mL of throughfall sample passed, was 5424 ± 449 copies for Cj1 and 4775 ± 204 copies for Cj2. Conversely, archaeal *amoA* gene copy numbers on 0.4- and 2-µm-pore filters were below the lower limit (<44 copies/reaction). Because 10-µm-pore filters used for throughfall of Cj1, in which nitrate production was observed (Fig. 2), contained the most abundant archaeal *amoA* genes, a clone library of archaeal *amoA* genes was constructed using 72 clones obtained from this filter (clone N2-throughfall). Phylogenetic analysis revealed that archaeal *amoA* gene sequences obtained from the throughfall of Cj1 mainly belonged to three groups (I, III, IV) of group I.1b (Fig. 4), although some sequences were clustered in a specific branch (group V) that was close to *Nitrosopumilus maritimus* (DQ085098), which is included in group I.1a.

Microbial DNA samples were also obtained from leaf samples of *C. japonica* (Cj1, Cj2, and Cj3). PCR led to recovery of partial archaeal *amoA* genes from all samples, whereas betaproteobacterial *amoA* genes were not recovered. Although archaeal *amoA* gene copy numbers of all leaf samples were below the quantitative lower limit, overall, 22 clones of the archaeal *amoA* gene were found in the microbial DNA samples collected from the leaf surfaces of Cj1 (clone name N2, six clones), Cj2 (clone name N3, nine clones), and Cj3 (clone name N1, seven clones). Phylogenetic analysis showed that all archaeal *amoA* gene sequences obtained from the tree leaves belonged to group I.1b (Fig. 4). The first group (I) included sequences from Cj1 and Cj3, whereas the second and third groups (II and IV) included those from Cj2.

## Discussion

### Microbial nitrification observed in throughfall from *C. japonica*

We verified that the ammonium concentrations in throughfalls of *C. japonica* were higher than those in rainfall (Fig. 2), indicating that tree canopies received atmospheric ammonia and aerosol ammonium. These results suggest the possibility that the tree phyllosphere is a habitat for some species of ammonia-oxidizing microbes and they could be washed into the throughfall. We found that the throughfall of *C. japonica* had microbial nitrification potential upon incubation of unfiltered throughfall samples. For the Cj1 throughfall, a clear increase in nitrate concentration that coincided with the decrease in ammonium and nitrite concentrations was observed in the unfiltered samples after 3- and 4-week incubation periods (Fig. 2). The amount of nitrate production exceeded the ammonium consumption during incubation, presumably because the dissolved organic nitrogen in the unfiltered throughfall sample underwent mineralization and subsequent nitrification. The nitrite concentration increased in samples that passed through 2- and 10-µm-pore filters, which may indicate that NOB could not pass through these filters. However, increases in nitrate concentration occurred to a lesser extent in unfiltered throughfall from Cj2. Although the cause for this difference was unclear, the chemical and microbiological conditions of Cj2 throughfall were not appropriate to exhibit its nitrification activity.

The δ^18^O values of nitrate in the unfiltered throughfall samples from Cj1 decreased markedly from +74.1 to +49.8 ‰ during 4 weeks of incubation (Fig. 3a, b). The δ^18^O value of nitrate produced by microbial activity should be much lower than that of the original nitrate in throughfall because of the incorporation of oxygen atoms from ambient water (δ^18^O: from −10 to −5 ‰) (Kendall 1998; Xue et al. 2009). During microbial nitrification, two-thirds of nitrate oxygen atoms are usually derived from water, whereas the other one-third originates from dissolved O_2_ (Snider et al. 2010). If the δ^18^O value of dissolved O_2_ is similar to that of atmospheric O_2_ (+23.5 ‰), the δ^18^O values of nitrate produced by microbial nitrification range from +1.2 to +4.5 ‰. Therefore, the isotopic mass balance calculation indicates that the amounts of nitrate produced by nitrification in the unfiltered throughfall sample after 4 weeks of incubation corresponded to 49.9–53.5 % of the original amount, which was consistent with the actual increase of nitrate from 219 to 332 µM, i.e., 51.8 % (Fig. 2). The δ^15^N value of nitrate in the unfiltered samples from Cj1 also decreased from +6.1 to −1.7 ‰ in response to 4 weeks of incubation (Fig. 3c, d). Garten (1992) reported that the δ^15^N values of ammonium were always lower than those of nitrate in throughfall, and generally <0 ‰ (Kendall 1998). Thus, the low δ^15^N values of ammonium were introduced into nitrate newly produced by nitrification during incubation, decreasing the δ^15^N values of nitrate in the unfiltered throughfall samples from Cj1.

We verified that the throughfall of *C. japonica* contained nitrifying microbes by detecting *amoA* genes, which participate in oxidation of ammonia. In the throughfall of *C. japonica* (Cj1 and Cj2), archaeal *amoA* genes were actually detected by PCR, although betaproteobacterial *amoA* genes were not found. Therefore, nitrification observed in the throughfall was probably accomplished by AOA. qPCR analysis estimated that the abundance of archaeal *amoA* gene in 1 mL of throughfall from Cj1 or Cj2 was <20 copies. This quantity was close to that of a low-nutrient groundwater sample (<100 copies mL^−1^, Reed et al. 2010), but lower than that of a deep oligotrophic lake sample (1.8 × 10^3^–5.6 × 10^3^, Auguet et al. 2012). Erguder et al. (2009) suggested that AOA play a more important role than AOB in the nitrogen cycle under low-ammonium, low-phosphate, low-pH, and/or sulfide-containing conditions. The initial ammonium concentrations in the throughfall of *C. japonica* (Cj1: 0.17 mM; Cj2: 0.40 mM) were as low as the level in which the moderately thermophilic AOA (*Candidatus* Nitrososphaera gargensis) showed high activity (0.14 or 0.79 mM) (Hatzenpichler et al. 2008). The phosphate concentrations were also low (<2 µM) in the throughfall. In addition, the pH of the throughfall was weakly acidic (Cj1: 5.7; Cj2: 5.6); therefore, the throughfall of *C. japonica* may be preferred by AOA rather than AOB. These results also suggest that the chemical and microbiological conditions in throughfalls might affect the variations in nitrification activities.

We confirmed that nitrate production and the decrease in δ^18^O and δ^15^N values of nitrate were reduced when the throughfall samples were filtered, particularly when 0.2-µm-pore filters that could eliminate a large portion of microbes were used (Figs. 2, 3a, c). In addition, nitrate production and the decrease in δ^18^O values of nitrate did not occur in rainfall samples, even when the unfiltered samples were incubated for 4 weeks (Fig. 2). Furthermore, neither archaeal nor betaproteobacterial *amoA* genes were detected in rainfall samples. These results suggest that the nitrification activity observed in unfiltered throughfall samples during incubation was caused by phyllospheric microbes washed off from the canopies rather than airborne microbes.

### Archaeal *amoA* genes recovered from the leaf surface of *C. japonica*

We verified the presence of ammonia oxidizers on the leaf surface of *C. japonica* by detecting functional genes. Archaeal *amoA* genes were recovered from microbes collected from the leaf surface of *C. japonica* (Cj1, Cj2, and Cj3) (Fig. 4). This is the first study to demonstrate the presence of archaeal *amoA* genes in a microbial community obtained from leaf surfaces. Although the existence of Crenarchaeota in several kinds of tree leaves was reported (Redford et al. 2010), the thaumarchaeotal distribution is never mentioned. In addition, we detected archaeal *amoA* genes in the leaf surface samples obtained from a Japanese cypress at the same sampling site (data not shown). Conversely, betaproteobacterial *amoA* genes were not detected from any leaf samples. These genetic features on the leaves were consistent with those observed in the throughfall; therefore, we consider AOA to be a substantial ammonia-oxidizer among microbes on tree canopies of *C. japonica*, even though archaea are generally recognized as minor members (<1 %) of the phyllospheric microbial community (Delmotte et al. 2009; Redford et al. 2010). Phylogenetic analysis of the archaeal *amoA* gene clones obtained from the microbes on tree leaves or in throughfall indicated that most of the archaeal *amoA* gene sequences belonged to group I.1b (Fig. 4). However the fifth group (V), to which some clones from the throughfall of Cj1 belonged, was very similar to a marine lineage represented by *Nitrosopumilus maritimus* that was included in group I.1a. Because there was no specificity to the clusters obtained from phyllosphere samples, it is likely that AOA on the tree leaves are not indigenous to the phyllosphere, but are instead ubiquitous in terrestrial environments.

The phyllosphere is a habitat available for certain species of microbes (e.g., Delmotte et al. 2009; Redford et al. 2010; Vorholt 2012). In this study, we showed that a subset of phyllospheric microbes from *C. japonica* has the potential for nitrification, although the approach used in this study was indirect (throughfall incubation). Another study also indicated that microbial nitrification occurred in the tree canopy based on analysis of the δ^18^O or ∆^17^O changes in nitrate throughout the rainfall, throughfall, and stemflow samples (Shi et al. 2014; Guerrieri et al. 2015). Because the total leaf surface area of terrestrial plants is as large as Earth’s terrestrial surface (Morris and Kinkel 2002), if microbial nitrification occurs on the foliar surfaces, it may play an important role in the global nitrogen cycle. We also found that AOA exist on leaves of *C. japonica* by detecting archaeal *amoA* genes, whereas betaproteobacterial *amoA* genes were not detected. Previous studies employing 16S rRNA gene pyrosequencing have indicated that AOB (e.g., *Nitrosospira*) were rarely detected from the tree phyllosphere bacterial community (Finkel et al. 2011, 2012). Conversely, the presence of AOB on tree leaves has been suggested in other studies in which the MPN and FISH techniques were used (Papen et al. 2002; Teuber et al. 2007). Additionally, it has been reported that epiphytic lichens (i.e., fungi and their algal symbionts) on foliar surfaces also contributed to nitrogen transformation (e.g., Gaige et al. 2007; Woods et al. 2012). Thus, further investigation is required to elucidate the community composition and activity of ammonia-oxidizing microbes in the phyllosphere.

## Conclusion

Our study examined the potential for microbial nitrification in the phyllosphere of tree canopies of *C. japonica* by incubation of the throughfall samples and detection of *amoA* genes. After incubation of unfiltered throughfall samples, nitrate production and decreased δ^18^O and δ^15^N values of nitrate were observed. These reactions were inhibited in sterile (filtered) throughfall samples. The archaeal *amoA* genes were detected from the throughfall and leaf surface of *C. japonica*. These results highlight the potential for microbial nitrification on tree canopies of *C. japonica*. Our findings indicate that the tree phyllosphere is a natural habitat for AOA and provide new insight into the biogeochemical nitrogen cycle.
